# Optimization of a multi-well colorimetric assay to determine haem species in *Plasmodium falciparum* in the presence of anti-malarials

**DOI:** 10.1186/s12936-015-0729-9

**Published:** 2015-06-24

**Authors:** Jill M. Combrinck, Kim Y. Fong, Liezl Gibhard, Peter J. Smith, David W. Wright, Timothy J. Egan

**Affiliations:** Division of Pharmacology, Department of Medicine, University of Cape Town, Observatory 7925, Cape Town, South Africa; Department of Chemistry, Vanderbilt University, Station B 351822, Nashville, TN 37235 USA; Department of Chemistry, University of Cape Town, Private Bag, Rondebosch, 7701 South Africa

**Keywords:** Malaria, *Plasmodium falciparum*, Haem, Haemozoin, β-haematin, Colorimetry, 24-well plate assay, Flow cytometry

## Abstract

**Background:**

The activity of several well-known anti-malarials, including chloroquine (CQ), is attributed to their ability to inhibit the formation of haemozoin (Hz) in the malaria parasite. The formation of inert Hz, or malaria pigment, from toxic haem acquired from the host red blood cell of the parasite during haemoglobin digestion represents a pathway essential for parasite survival. Inhibition of this critical pathway therefore remains a desirable target for novel anti-malarials. A recent publication described the results of a haem fractionation assay used to directly determine haemoglobin, free haem and Hz in *Plasmodium falciparum* inoculated with CQ. CQ was shown to cause a dose-dependent increase in cellular-free haem that was correlated with decreased parasite survival. The method provided valuable information but was limited due to its low throughput and high demand on parasite starting material. Here, this haem fractionation assay has been successfully adapted to a higher throughput method in 24-well plates, significantly reducing lead times and starting material volumes.

**Methods:**

All major haem species in *P. falciparum* trophozoites, isolated through a series of cellular fractionation steps were determined spectrophotometrically in aqueous pyridine (5 % v/v, pH 7.5) as a low spin complex with haematin. Cell counts were determined using a haemocytometer and a rapid novel fluorescent flow cytometry method.

**Results:**

A higher throughput haem fractionation assay in 24-well plates, containing at most ten million trophozoites was validated against the original published method using CQ and its robustness was confirmed. It provided a minimum six-fold improvement in productivity and 24-fold reduction in starting material volume. The assay was successfully applied to amodiaquine (AQ), which was shown to inhibit Hz formation, while the antifolate pyrimethamine (PYR) and the mitochondrial electron transporter inhibitor atovaquone (Atov) demonstrated no increase in toxic cellular free haem.

**Conclusions:**

This higher throughput cellular haem fractionation assay can easily be applied to novel anti-malarials with a significantly decreased lead time, providing a valuable tool with which to probe the mechanisms of action of both new and established anti-malarials.

## Background

The inhibition of the haem detoxification pathway in the malaria parasite, *Plasmodium falciparum* remains an attractive drug target with several important anti-malarials, primarily quinolines proposed to target it [1]. During its asexual erythrocytic cycle, free haem, derived from the digestion of host red blood cell haemoglobin (62 ± 13 − %) is autoxidized to poisonous haematin (Fe(III)PPIX), predominantly in the metabolically active trophozoite [2]. In an early study Ginsburg et al. detected an increase in membrane associated haem upon CQ and amodiaquine (AQ) treatment of cultured *P. falciparum*, but recovery of haemozoin in drug-free controls in this study was very low, with only 20–30 % detected [3]. In a subsequent study it was shown that the overwhelming majority of this released haem (up to 95 %) is efficiently sequestered into inert crystalline haemozoin (Hz) within the parasite’s acidic digestive vacuole. Transmission electron microscopy (TEM) with electron energy loss spectroscopy (EELS) used to map the distribution of elemental Fe in mature trophozoite-infected erythrocytes (chloroquine (CQ) sensitive, D10 strain), confirmed Hz associated Fe is concentrated in the acidic digestive vacuole with very little Fe in the cytosol [2]. Hz formation has been shown to be closely associated with lipids [4]. Recently, neutral lipids associated with Hz were shown to mediate β-haematin (BH, synthetic Hz) formation under physiological conditions at rates analogous to parasite Hz formation [5]. This process of cellular lipid mediated Hz formation is mimicked synthetically in a detergent-mediated assay which substitutes neutral lipids for the lipophilic detergent NP-40 [6]. This assay has been applied as a high throughput screening tool and identified several novel scaffolds capable of inhibiting Hz formation [7, 8]. The Hz inhibition pathway remains a favourable drug target despite resistance to known quinolines and related drugs which have been attributed to mutations in the parasite’s ‘chloroquine resistance transporter’ (PfCRT) and other digestive vacuole membrane proteins [9]. In comparison to CQ-sensitive strains (CQS), resistant parasites have been shown to accumulate up to ten times less CQ in the digestive vacuole, falling outside the effective therapeutic range for CQ [10]. The formation of Hz is unaffected by PfCRT mutations and so Hz inhibitors remain one of the most successful classes of anti-malarials to date.

The use of detergent-based screening methods as a synthetic measure of the ability of a compound to inhibit the formation of BH is routinely used to identify BH inhibitors [8, 11]. This method has been translated into an analogous process in malaria parasites cultured in vitro, the haem fractionation assay, a method capable of directly measuring haem species in CQS *P. falciparum* [12]. Originally applied to CQ, this cellular fractionation technique colorimetrically measures haem as Fe(III)haem-pyridine complex based on a previously published method by Ncokazi and Egan in which it was shown that in a mixture of BH and haematin, the latter forms a low-spin complex with aqueous pyridine (5 % v/v, pH 7.5) without disturbing BH [13]. Using the cellular haem fractionation assay, CQ was shown to cause a dose-dependent increase in ‘free’ haem (i.e., labile haem that can be solubilized with detergent), along with a decrease in Hz correlated to the survival of *P. falciparum* cells. TEM with EELS of CQ-treated cells showed a redistribution of Fe from the digestive vacuole into the parasite cytoplasm [12]. The method provided valuable information, successfully demonstrating the ability of CQ to inhibit cellular Hz formation. Due to its protracted nature, however, it could not easily be extended to other drugs. Here, a modified technique designed to increase output and reduce material costs is described in detail. Performed in 24-well plates, multiple drug concentrations can be evaluated in one session with relatively low amounts of parasite starting material. At minimum results can be obtained for two compounds per week, a significant improvement in output over the previous method, which produced results for one compound every 2 months. The method was successfully validated against the original haem fractionation assay performed in flasks using CQ. This validated method was applied to three clinically relevant anti-malarials covering a broad spectrum of mechanisms of actions: the 4-aminoquinoline AQ which like CQ has been shown to inhibit BH formation, the non-BH inhibiting antifolate pyrimethamine (PYR) [7] and finally the non-BH inhibiting anti-malarial atovaquone (Atov). While initial results for Atov showed an unanticipated increase in the percentage of free haem with increasing Atov concentration, the total amount of haem iron per cell was found to be significantly less in cells treated with Atov. This corresponds to unchanged free haem per cell with increasing Atov concentration. This example with Atov demonstrates the importance of establishing the total amount of haem iron per cell, a calculation which requires the determination of the cell count in each well of the 24-well plate. Cell counts were determined using a haemocytometer and flow cytometry based method with fluorescent cell staining [14–19]. Acquiring cell counts for a large number of samples with a haemocytometer proved a tedious process, as a result counting was also performed with a novel flow cytometry method using SYBR green I fluorescent staining of isolated cells. Over the course of the 48 h lifecycle of the parasite the amount of DNA increases as the parasite matures from early rings to multi-nucleated schizonts, translating to an increase in the SYBR green I fluorescence signal as a result of binding to double stranded parasite DNA [19]. SYBR green I was specifically chosen due to its low binding affinity for RNA and preference for double stranded DNA over single stranded DNA [20]. In addition to relieving the burden of manual cell counting, this flow cytometry method can also provide important information about morphological changes in the presence of cells treated with anti-malarials.

The method described here is a modified version of an established technique which details the increased output of a previously published assay allowing for the determination of haem species spectroscopically in isolated *P. falciparum* trophozoites.

## Methods

Unless otherwise stated, all materials used were obtained from Sigma Life Sciences.

### In vitro culture of *Plasmodium falciparum* parasites

A CQS strain of *P. falciparum*, D10, was used throughout this study and cultured using a modified method of Trager and Jensen [21]. Briefly, parasites were maintained in fresh type O+ human erythrocytes in medium containing 10.4 g/l RPMI 1640 with glutamine (and without NaHCO_3_), 4 g/l glucose, 6 g/l HEPES, 0.088 g/l hypoxanthine, 1.2 ml/l gentamycin and 5 g/l Albumax II (GIBCO). Directly before use, 8.4 ml of 5 % (w/v) NaHCO_3_ was added to 200 ml medium. Cultures were kept at 37 °C in 200-ml flat-bottom flasks under a gas environment of 3 % O_2_, 4 % CO_2_, and N_2_. The integrity of the culture was monitored daily using 10 % (v/v) Giemsa (Merck) stained slides. Synchronization was maintained by treating with five volumes of 5 % (w/v) sorbitol for 10 min on ring days. Post synchronisation, cultures were centrifuged at 750 rcf for 5 min and the layer of debris corresponding to lysed cells and the associated Hz was carefully removed from the culture by aspiration.

### Biological IC_50_ determination

In vitro antiplasmodial activity was determined via the parasite lactate dehydrogenase assay using a modified method described by Makler et al., which colorimetrically measures *Plasmodium* lactate dehydrogenase [22]. Test samples were prepared to a 20 mg/ml stock solution in 100 % dimethyl sulfoxide (DMSO, Merck) and stored at −20 °C. On the day of use, samples were diluted in medium to a starting concentration of 100 μg/ml and serially diluted two-fold in medium in 96-well plates. All samples were tested in triplicate at ten concentrations, with the lowest concentration being 0.2 μg/ml. The highest concentration of DMSO to which the parasites were exposed was 0.5 % (v/v). The IC_50_-values were obtained using a non-linear dose–response curve fitting analysis via Graph Pad Prism v.4.0 software [23].

### Haem fractionation assay

#### Incubation and harvesting

Aliquots of 2 ml of early ring-stage sorbitol synchronized parasites at 5 % parasitaemia and 2 % haematocrit were added to each well of a 24-well flat-bottom cell culture plate (Greiner Bio-One). All test compounds were diluted in medium from the previously prepared 20 mg/ml stock solution and added to the plate such that the final volume per well was 2020 μl and the final concentrations tested were at several multiples of the compound’s IC_50_-value. Each concentration was tested as four replicates and the highest concentration of drug tested was at 4 × IC_50_, since beyond this concentration very few cells remained for harvesting. Plates were incubated at 37 °C in a gas chamber with 3 % O_2_, 4 % CO_2_, and N_2._ After 32 h, excess supernatant was aspirated and Giemsa-stained thin smears were prepared on glass slides at each concentration tested. Each pellet was resuspended in 1.9 ml phosphate buffered saline (PBS) pH 7.5 and transferred to a deep well 2.2 ml rectangular 96-well plate (Labcon). The new plates ensured maximum sample recovery in subsequent steps and were essential for further processing. Erythrocytes were lysed with the addition of 100 μl 1 % (w/v) saponin. After 2 min at 37 °C plates were centrifuged at 750 rcf for 15 min. The supernatant was aspirated and the pellets washed three times with 0.5 ml PBS to remove all traces of erythrocyte haemoglobin (Hb). The washed pellet, corresponding primarily to isolated trophozoites, was resuspended, diluted to a final volume of 100 μl with PBS pH 7.5 and accurately transferred to a round-bottomed, 96-well 0.5 ml plate (Axygen Scientific) referred to as the stock plate. The stock plate was used to prepare a second plate for cell counting after which it was frozen at −80 °C.

#### Preparation of the counting plate and cell fixation

A counting plate was prepared by adding 10 μl of the isolated washed trophozoites to the corresponding wells of a flat-bottomed 96-well plate. The cells were fixed with 0.125 % (v/v) glutaraldehyde in PBS pH 7.5 to a final volume of 200 μl and refrigerated at 4 °C overnight.

#### Haemocytometer counting

Haemocytometer counting was performed on the first row of each plate only, corresponding to six samples, each at a different concentration of the drug tested. The haemocytometer determined cell counts for these six samples were statistically compared to the cell counts determined by flow cytometry to test for agreement. Ten μl of cells from the counting plate were loaded onto a bright-line haemocytometer and five large squares were counted after 10 min of settling. The concentration of cells in each well of the plate was determined using equation 1:

1 $$ {C}_H = N\times F\times DF\times 10, 000 $$

Where:

*C*_*H*_ = concentration of cells per ml as determined with haemocytometer

*N* = number of cells counted in five fields

*F* = number of fields counted = 5

*DF* = dilution factor

#### Flow cytometry counting

Cell counts for all samples on the counting plate were determined using flow cytometry [14, 16, 17]. Samples were analysed on a Becton Dickinson FACSCalibur using SSC/FL1_530nm_ with CellQuestPro software. Typically 10,000 events were counted for each sample. Samples were prepared by diluting 100 μl of cells from the counting plate with 800 μl of 1 × SYBR® green I in PBS pH 7.5 and incubating for 30 min in the dark at 37 °C. Next, each sample was spiked with 100 μl of Trucount™ beads (Becton Dickinson) such that each sample contained a known fixed amount of fluorescent beads in a final volume of 1 ml and was mixed well. The concentration of cells in the acquisition tube was calculated according to equation 2:

2 $$ {C}_F=\left(T/B\right)\times {C}_B $$

Where:

*C*_*F*_ = concentration of cells per ml as determined with flow cytometry

T = number of trophozoites gated

*B* = number of fluorescent beads gated

*C*_*B*_ = concentration of fluorescent beads in the acquisition tube per ml (calibrated bead count per acquisition is unique to each lot of tubes obtained from the supplier)

**Fig. 1:**
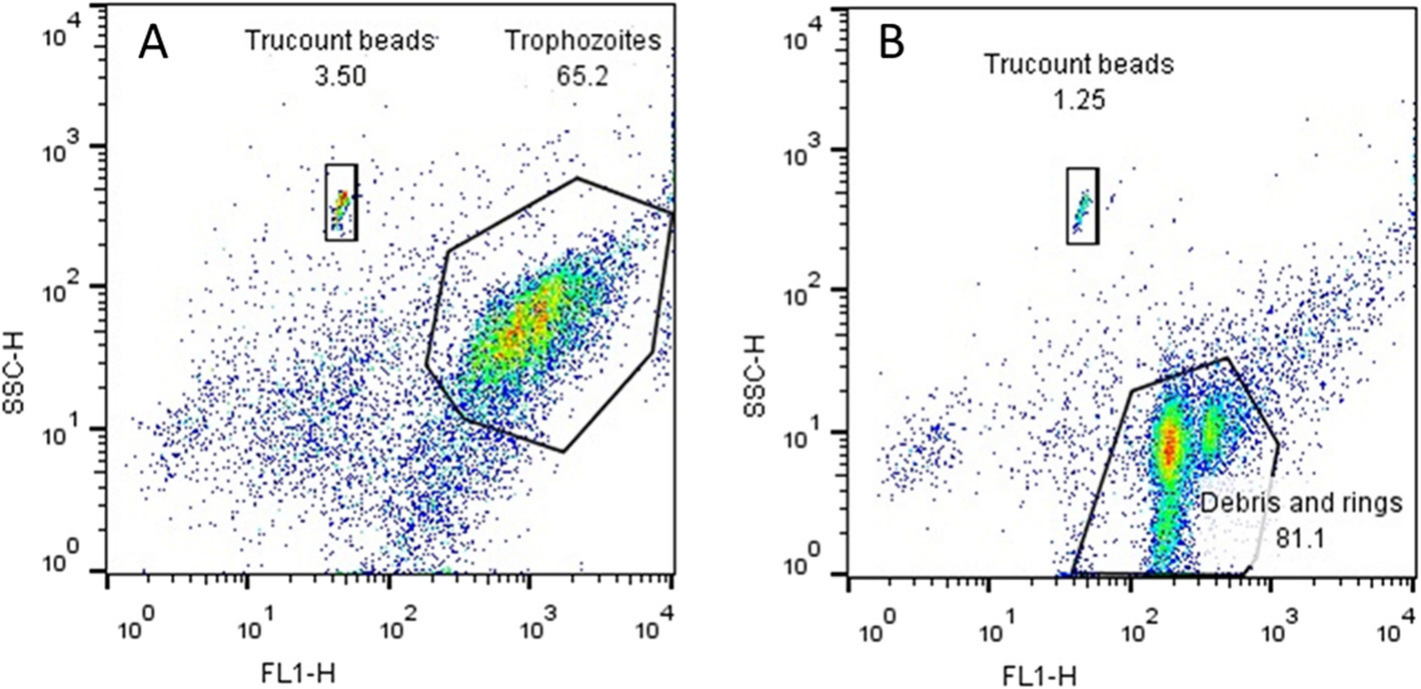
Dot plots displaying isolated *Plasmodium falciparum* cells corresponding to trophozoites (**a**) and rings and cell debris (**b**) after staining with the fluorescent DNA dye, SYBR green I and the populations corresponding to fluorescent Trucount beads

Trophozoite gates were established by running isolated trophozoites obtained from a highly synchronous trophozoite culture through the process of harvesting and flow cytometry analysis as described above. The resulting dot plot in Fig. 1a represents a population corresponding to primarily trophozoites. The presence of trophozoites was confirmed with a 10 % (v/v) Giemsa-stained slide. The same process was applied to a highly synchronous ring culture and the resulting dot plot of cell material accrued as a consequence of the harvesting process (most likely corresponding to both rings and cell debris) can be seen in Fig. 1b. A 10 % (v/v) Giemsa-stained slide confirmed the presence of accumulated cell debris; however it was difficult to distinguish rings from the debris. The trophozoite population corresponds to cells of a much higher fluorescent intensity and side scatter (SSC) indicating more complex cells with more nucleic acid in comparison to the population of rings and debris with lower fluorescent intensity and SSC. The latter population was omitted during gating and only intact trophozoites were gated and used to determine cell counts. The population of cells corresponding to Fig. 1a and b were assayed for haem as described below in the haem fraction assay. Initial gating was performed with unstained cells to account for autofluorescence. All samples were analysed using FloJo software version 10 (Tree Star Inc).

#### Haem fractionation

After thawing the stock plate, 50 μl of water was added and the plate was sonicated for 5 min in an ultrasound bath (53 kHz, 320 W, Bandelin Sonerex). This was followed by the addition of 50 μl each of 0.2 M HEPES buffer pH 7.5 and water. The plate was then centrifuged at 3600 rpm for 20 min. Very carefully, without disturbing the pellet, the supernatant was transferred to an adjacent set of wells on the same plate. The supernatant was processed further with 50 μl 4 % SDS, sonicated for 5 min and then incubated at 37 °C for 30 min. Fifty μl of 0.3 M NaCl and 50 μl of 25 % (v/v) pyridine in 0.2 M HEPES pH 7.5 were added and 200 μl of the solution was transferred to a flat-bottomed UV-Star 96-well plate. This fraction corresponds to the Hb fraction.

The pellet was treated with 50 μl water, 50 μl 4 % SDS and resuspended. The plate was sonicated for 5 min and incubated at 37 °C for 30 min to solubilize free haem. This was followed by the addition of 50 μl 0.2 M HEPES pH 7.5, 50 μl 0.3 M NaCl and 50 μl 25 % pyridine. The plate was centrifuged at 3700 rpm for 20 min. Very carefully without disturbing the pellet, the supernatant was transferred to an adjacent set of wells on the same plate. The supernatant was diluted to a final volume of 400 μl with water. This fraction corresponds to the free haem fraction; 200 μl of this solution was transferred to the flat-bottomed UV-Star 96-well plate, the same plate previously used for the Hb fraction.

**Fig. 2:**
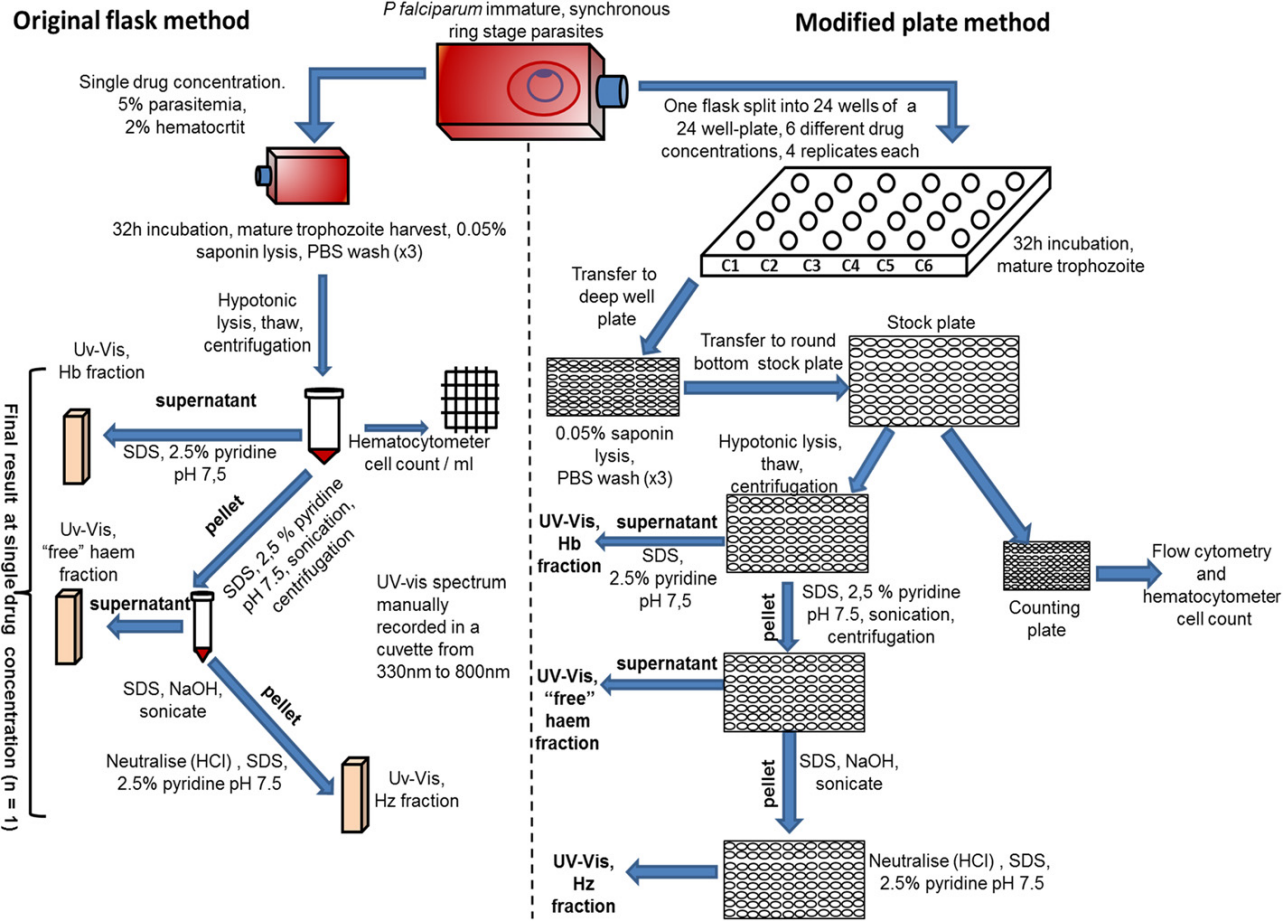
Flow diagram showing the haem fractionation assay: original flask method (*left*) versus increased throughput plate method (*right*). The haem fractionation assay is a cellular fractionation technique based on the ability of neutral aqueous pyridine to selectively form a low spin haem-pyridine complex with free haem in the presence of Hz [13]. The original method was performed in 250 ml culture flasks, testing a single drug concentration at a time and performing spectroscopic measurements in a cuvette [12]. The modified method was performed in 24-well plates, testing several drug concentrations, four replicates at a time and using a multiwell plate reader to record absorbance, resulting in a six-fold increase in output. Immature ring stage parasitized red blood cells were inoculated with several different increasing drug concentrations. After 32 h, mature trophozoites were harvested via saponin lysis of erythrocytes. Following hypotonic lysis and centrifugation, SDS treated soluble Hb was measured in the supernatant as a low spin haem-pyridine complex. The pellet was further treated with SDS and pyridine to solubilize free haem, measured in the supernatant following centrifugation. Hz was measured after solubilizing the pellet in NaOH, neutralizing with HCl and treating with pyridine

The remaining pellet containing Hz was solubilized in 50 μl of water and 50 μl 0.3 M NaOH. The plate was sonicated for 15 min and incubated at 37 °C for 30 min and 50 μl each of 0.2 M HEPES pH 7.5, 0.3 M HCl and 25 % pyridine was added followed by 150 μl water. This fraction corresponds to the Hz fraction; 200 μl of this solution was transferred to vacant wells in the flat-bottomed UV-Star 96-well plate containing the Hb and free haem fractions. The UV-visible spectra of haem as Fe(III)haem-pyridine complex was recorded using a multi-well plate reader (Spectramax 340PC, Molecular Devices). The absorbance maxima of the Fe(III)haem pyridine complex in each well was used to calculate the percentage of each haem species in each sample as the final volume for each fraction was identical. The procedure is presented graphically in Fig. 2.

#### Haem standard curve

The total amount of haem in each fraction was quantified using a haem standard curve prepared from a 100 μg/ml haem standard solution of haematin (porcine) in 0.3 M NaOH. Serial dilutions of the standard were carried out in a 96-well plate with 100 μl 0.3 M NaOH as a blank. Fifty μl of each of the following solutions were added to 100 μl of haematin standard: 0.2 M HEPES pH 7.5, 4 % (w/v) SDS, 0.3 M NaCl, 0.3 M HCl, 25 % pyridine in 0.2 M HEPES pH 7.5 and water. The visible spectra of haem as Fe(III)haem-pyridine complex were recorded in a multi-well plate reader. The amount of haem Fe per cell was calculated by dividing the total haem Fe in each fraction by the number of cells determined in each fraction.

#### Statistical analysis

Bland Altman analysis was used to assess the agreement between the original haem fractionation method and modified plate method [24]. For determination of statistical significance of differences in measurements relative to controls, a two-tailed *t*-test (95 % CI) was used and is displayed on graphs using asterisks (* P <0.05; ** P <0.01; *** P <0.001). In all cases data represent a minimum of three repeats. All analysis was carried out using GraphPad Prism version 4.0 software [23].

## Results and discussion

### Cell counting: haemocytometer versus flow cytometer

**Fig. 3:**
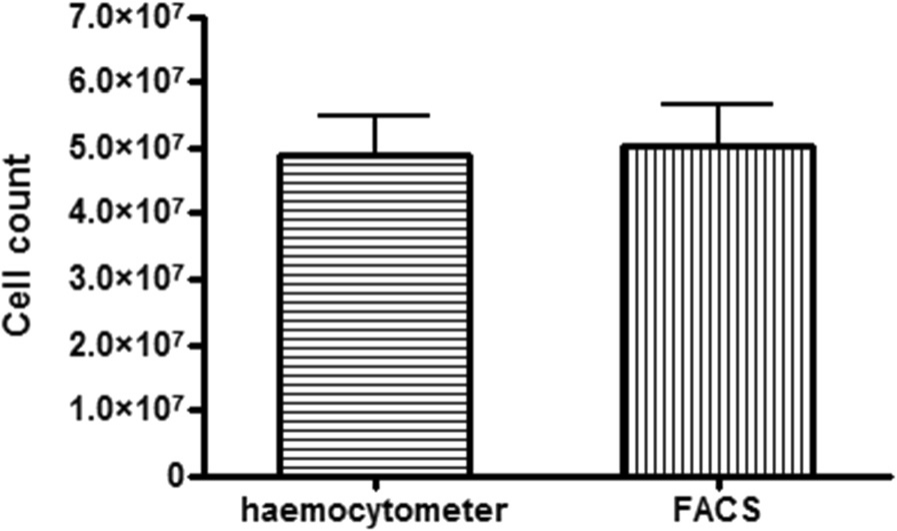
Average counts obtained using a haemocytometer (*horizontal stripes*) and FACS (*vertical stripes*) for controls in each experiment. The mean count obtained using the haemocytometer did not differ significantly from the mean count obtained using the FACS method (*n* = 6, 6), *P* = 0.876 (unpaired two-tailed *t*-test, 95 % CI)

Cell counts, required for the determination of the total amount of haem Fe per cell (reported in fg/cell) were originally determined manually using a haemocytometer. This method was found to be slow and tedious when applied to 24 samples per plate and an alternative, more efficient method for counting cells was set up using flow cytometry. Post fixation with glutaraldehyde, cells were stained with the DNA binding fluorescent dye SYBR green I, spiked with a fixed number of fluorescent Trucount™ beads (Becton Dickinson) and analysed with a Becton Dickinson FACS Calibur using SSC/FL1_530nm_ with CellQuestPro software. Cell counts using flow cytometry were performed on all wells of each 24-well plate and haemocytometer counts were routinely done on the first row only of each 24-well plate for comparison and to establish agreement between the methods. In both cases only intact isolated trophozoites were counted. Flow cytometry is a faster, less subjective process than haemocytometer counting but is not without limitations. The first issue relates to the accurate gating of cells. Despite having established a gate specifically for trophozoites (Fig. 1a) which excludes any rings and cell debris collected during the process of harvesting (Fig. 1b), an overlap exists where cells which appear as larger, mature rings have the same SSC and fluorescent intensity as immature or underdeveloped trophozoites and it is not possible to distinguish the two, which can result in an inaccurate trophozoite cell count. The second issue is counting aggregates of cells as a single complex cell resulting in undercounting, however this is largely overcome by mixing samples well during preparation and running the samples at a low flow rate. Despite the limitations associated with both counting methods, it was found there was no statistical difference between the means as determined by each counting method (unpaired two-tailed *t*-test, 95 % CI) for the untreated control (Fig. 3). Furthermore, no significant difference was seen at any concentration of the four drugs tested (Table 1).

**Table 1:** Summary of *P*-values (unpaired 2-tailed *t*-test, 95 % CI) obtained when comparing the counts obtained with the FACS method to the haemocytometer method at each concentration of each drug tested

CQ (nM)	*P*	AQ (nM)	*P*	Atov (nM)	*P*	PYR (nM)	*P*
7.5	0.92	12.5	0.12	1	0.75	25	0.13
15	0.68	25	0.56	2	0.92	50	0.12
30	0.32	50	0.056	4	0.69	100	0.087
45	0.89	75	0.28	8	0.83	150	0.11
90	0.96	100	0.061	16	0.23	200	0.86

In all cases the mean count obtained using the haemocytometer did not differ significantly from the mean count obtained using the FACS method

### Assessment of the robustness of the assay

In the previous report on the flask method for determination of haem species in *P. falciparum*, the method was not described in detail [12]. In particular, the specificity of the assay for different haem species in the various fractions was not investigated with respect to cross-contamination at each of the fractionation steps. This has now been addressed in the plate assay.

**Fig. 4:**
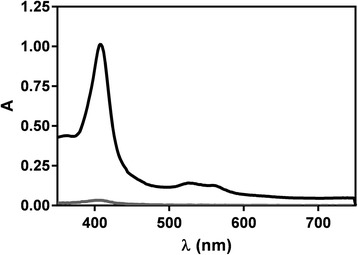
Absorbance spectra of Hz haem for the ring (*gray*) and trophozoite (*black*) stages obtained from the same culture. The Soret peak obtained from the ring stage is very weak compared to that from the trophozoite stage. The maximum content of haemozoin in the ring culture is 4.2 ± 0.4 % (*n* = 4) of that in the trophozoites. In order to detect a significant signal from the ring stage, these experiments were conducted with 2.5 × 10^7^ parasites

When cultured parasites are synchronized with sorbitol, trophozoites present in the asynchronous culture are lysed and release their haemozoin. If no precautions were taken, this haemozoin would be carried into the synchronized culture used in the assay. To avoid this, visible pigmented cell debris was removed by aspiration immediately following sorbitolization. To confirm that significant quantities of pre-formed haemozoin did not contaminate the sample, a synchronous culture was subjected to the process of harvesting, followed by the haem fractionation assay at the ring stage soon after sorbitol treatment. At this stage of the lifecycle, early rings would have produced no haemozoin. Consequently, any haem detected in the haemozoin fraction arises from either haemozoin carried over during synchronization, or from a small fraction of trophozoites that survive sorbitol treatment. Figure 4 shows that the absorbance spectrum of haem present in this fraction was very low compared to material isolated from the same culture at the trophozoite stage. The cross contamination from previous cycles was thus at most 4 % (since a portion of the small absorbance seen almost certainly arose from trophozoites still present in the culture post synchronization). Thus, the carry-over of Hz from previous cycles was found to be negligible.

**Fig. 5:**
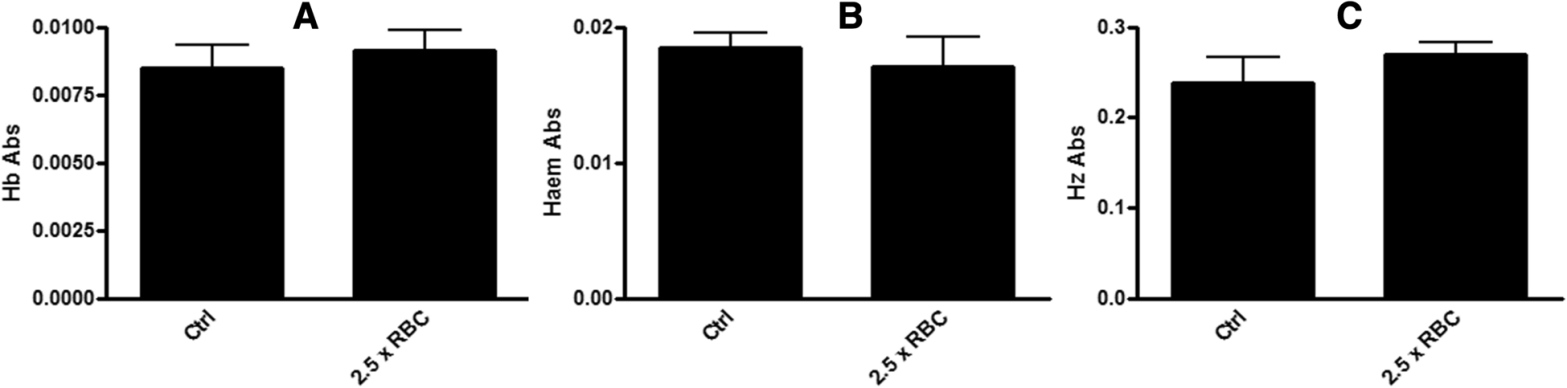
The Soret band absorbance readings for Hb (**a**), haem (**b**) and Hz (**c**) fractions of a sample spiked with 100 μl of RBCs compared to a control sample containing 40 μl of 5 % parasitized RBCs under identical conditions. No significant change was seen in the haem levels of the Hb fraction (*P* = 0.57), the haem fraction (*P* = 0.52) or the Hz fraction (*P* = 0.33) of the sample spiked with 2.5 fold more RBCs than the control (unpaired two-tailed *t*-test, 95 % CI, *n* = 6). Both the control and spiked sample contained 10^7^ parasites and were from the same culture

A second potential source of cross-contamination is RBC Hb, which is the dominant form of haem in the total culture at 5 % parasitaemia. Trophozoites isolated by saponin lysis are accompanied by lysed RBC membranes. The pellet could be contaminated with Hb if the washing steps are not efficient enough to remove it and would result in elevated trophozoite Hb readings. The elevated readings could potentially carry over into subsequent fractions. To test this, the 40 μl RBC pellet containing 5 % parasitized cells was spiked with 100 μL of packed RBCs. This mixture was then subjected to the usual harvesting and haem fractionation assay and all the haem species were measured. As shown in Fig. 5, none of the fractions showed any significant change in haem levels relative to the unspiked control. This demonstrated that cross-contamination by RBC Hb is not significant provided that the washing protocol is followed.

Finally, the ‘free’ haem fraction could potentially be contaminated as a result of the breakdown of haemozoin during ultrasound treatment in the presence of SDS. Two approaches were used to test this. Firstly, the duration of ultrasound treatment was increased three-fold to 15 min. No significant change in free haem levels was detected (Table 2). Secondly, the pellet containing ‘free’ haem and haemozoin was spiked with isolated haemozoin prior to addition of SDS and ultrasound treatment. The haemozoin was obtained from the final pellet of a control experiment isolated prior to addition of NaOH and doubled the quantity of haemozoin in the sample. Isolation of ‘free’ haem from this spiked sample showed no significant difference relative to an unspiked control (Table 2). This confirmed that haemozoin does not contaminate the ‘free’ haem fraction.

**Table 2:** Summary of mean absorbances (*n* = 6) and *P*-values (unpaired 2-tailed *t*-test, 95 % CI) for the control, a sample sonicated for 15 min and a sample spiked with Hz

Sample	Average absorbance	*P*-value
Control	0.024 ± 0.003	N/A
Sonicated for 15 min	0.021 ± 0.001	0.08
Hz Spiked	0.027 ± 0.006	0.26

### Validation of plate method with CQ

**Fig. 6:**
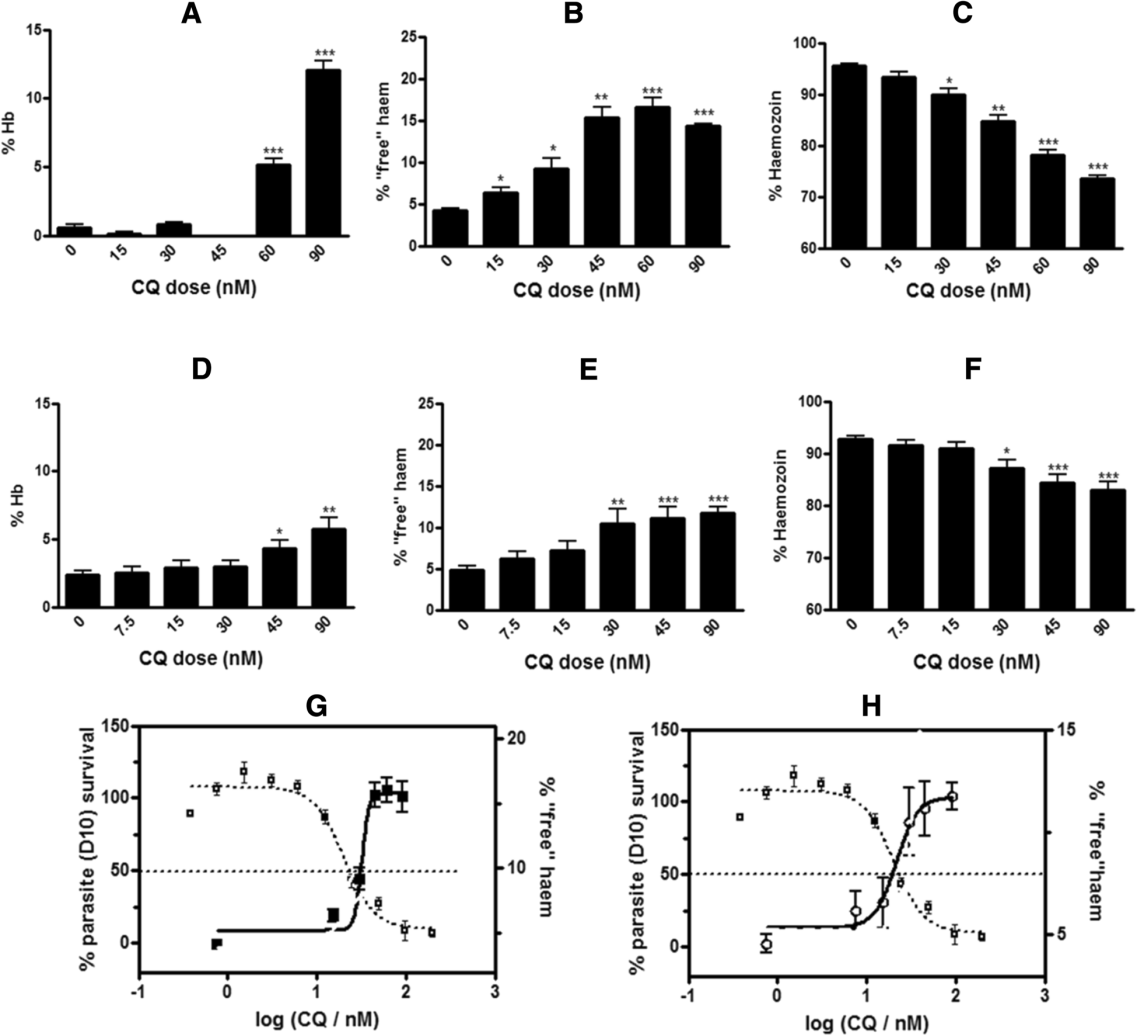
Percentage of haem species found in chloroquine-treated trophozoites assayed with the original flask method (**a**, **b**, **c** and **g**) and the new plate method (**d**, **e**, **f** and **h**) Hb (**a**, **d**), ‘free’ haem (**b**, **e**) and Hz (**c**, **f**) at increasing concentrations of CQ are expressed as a percentage of the total haem found in isolated trophozoites. Statistical significance, calculated using a two-tailed *t*-test (*error bars* showing 95 % CI) is expressed relative to the control using *asterisks*. The parasite survival curve (left axis) was determined using the parasite lactate dehydrogenase assay [22] and is overlaid with the percentage of ‘free’ haem (right axis) determined by both the original flask method (**g**) and new plate method (**h**) as a function of CQ concentration. Plots are scaled so that the *dotted line* in this and subsequent figures corresponds to both the parasite growth inhibition IC_50_ as determined by the parasite lactate dehydrogenase assay and the *midpoint* of the ‘free’ haem increase determined using the haem fractionation assay. CQ IC_50_ in D10 = 19.2 ± 4.2 nM

The plate method was validated in CQS D10 *P. falciparum* inoculated with CQ, since a full dose response profile was previously reported for CQ using the flask method at several different concentrations [12], allowing for direct comparison of results. The effect on all three haem species was examined and statistically compared to the previously published data obtained with the original flask method (Fig. 6a, b, c, and g) [12].

Both methods showed a dose dependent increase in percentage ‘free’ haem with corresponding decrease in Hz in CQS *P. falciparum* inoculated with CQ (Fig. 6b, c, e and f). In both cases the rise in ‘free’ haem mirrors the decline in the survival curve of the parasite such that at higher concentrations of ‘free’ haem corresponding to parasites exposed to high doses of CQ very few trophozoites survive after the 32-h incubation period (Fig. 5g, h). Although the trend for both methods is the same, the modified plate method showed a more muted response for both the increase in ‘free’ haem and decrease in Hz (Fig. 6b, c, e and f). In the flask method, the maximum increase in ‘free’ haem was 10.2 % compared to the control, while in the plate method the maximum increase in ‘free’ haem compared to the control was 7.0 %. This is probably attributable to the decreased absorbance as a result of reduced starting material volumes for each sample, which is in turn associated with larger errors. Small but significant sample losses which occur at each fractionation step, as well as sample carry over during the transfer and separation of the supernatant and pellets are also likely contributory factors.

**Fig. 7:**
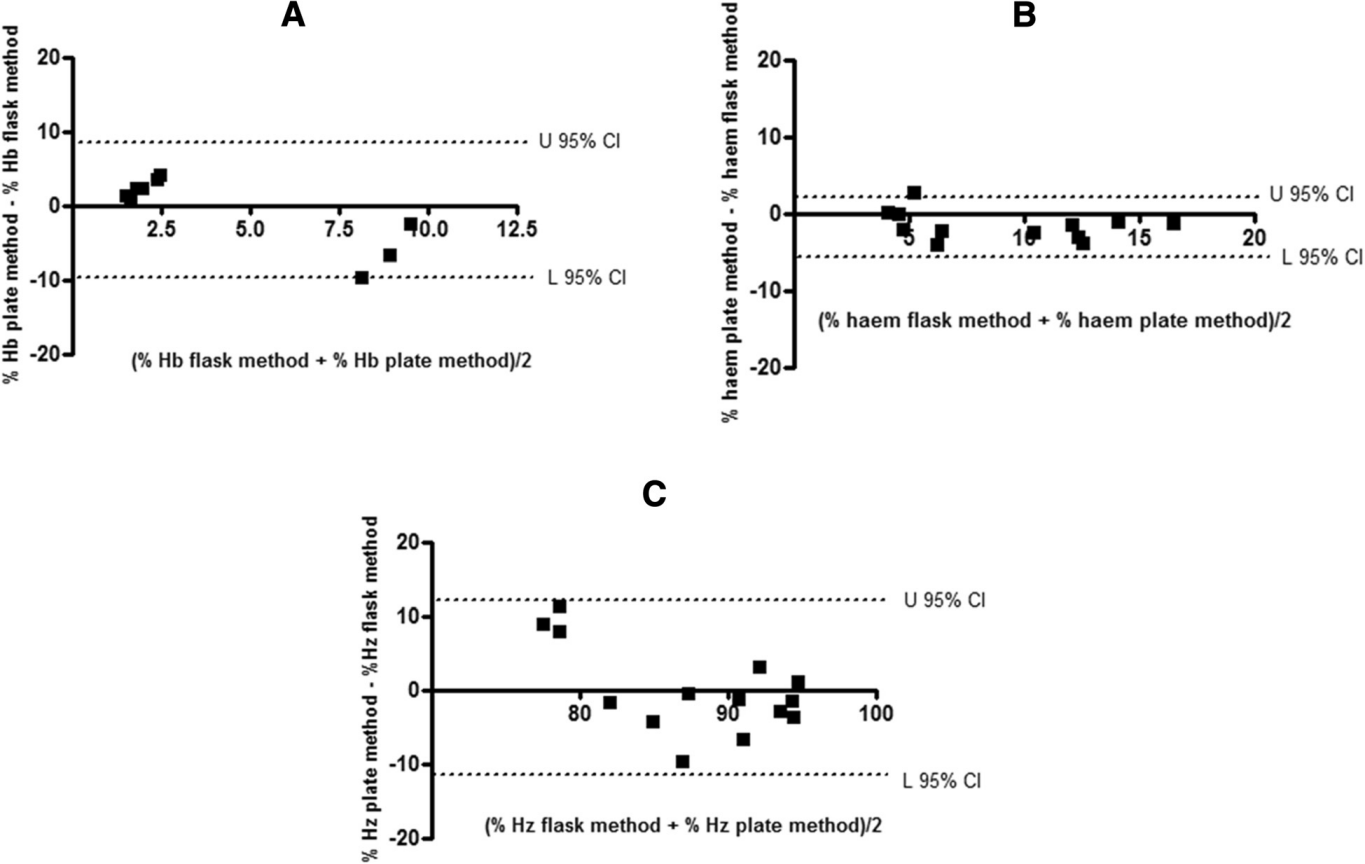
Bland Altman plots displaying the differences between the results obtained with the plate method and the flask method at each measurement for Hb (**a**), ‘free’ haem (**b**) and Hz (**c**). The *dotted lines* represent the upper and lower 95 % limits of agreement between the two methods. Outliers were removed using the modified Thompson Tau test before analysis. Data are representative of three separate experiments

Bland Altman analysis was used to statistically compare the two methods with respect to the percentage of each haem species calculated [24]. For each haem species the difference in the average of the result obtained with the flask and modified plate method was plotted against the mean of the result obtained with each method. The agreement between the two methods for both ‘free’ haem (Fig. 7b) and Hz (Fig. 7c) were good, showing narrow limits of agreement in comparison to the means. Hz however does show evidence of systematic error for the three lowest mean percentages, corresponding to cells which were dosed at 90 nM CQ (greater than four times the IC_50_ of CQ). This is most likely associated with larger errors due to poor recovery at high drug concentrations. Hb (Fig. 7a) showed much broader limits of agreement between the methods relative to the mean for each set of measurements. The determination of Hb, specifically in the plate method was very often associated with large errors owing to the small sample volume used, resulting in low absorbance values for Hb, which is present in very small amounts in mature trophozoites. In view of the good agreement for Hz and especially ‘free’ haem, the higher throughput plate method was subsequently used for further investigations of its versatility.

**Fig. 8:**
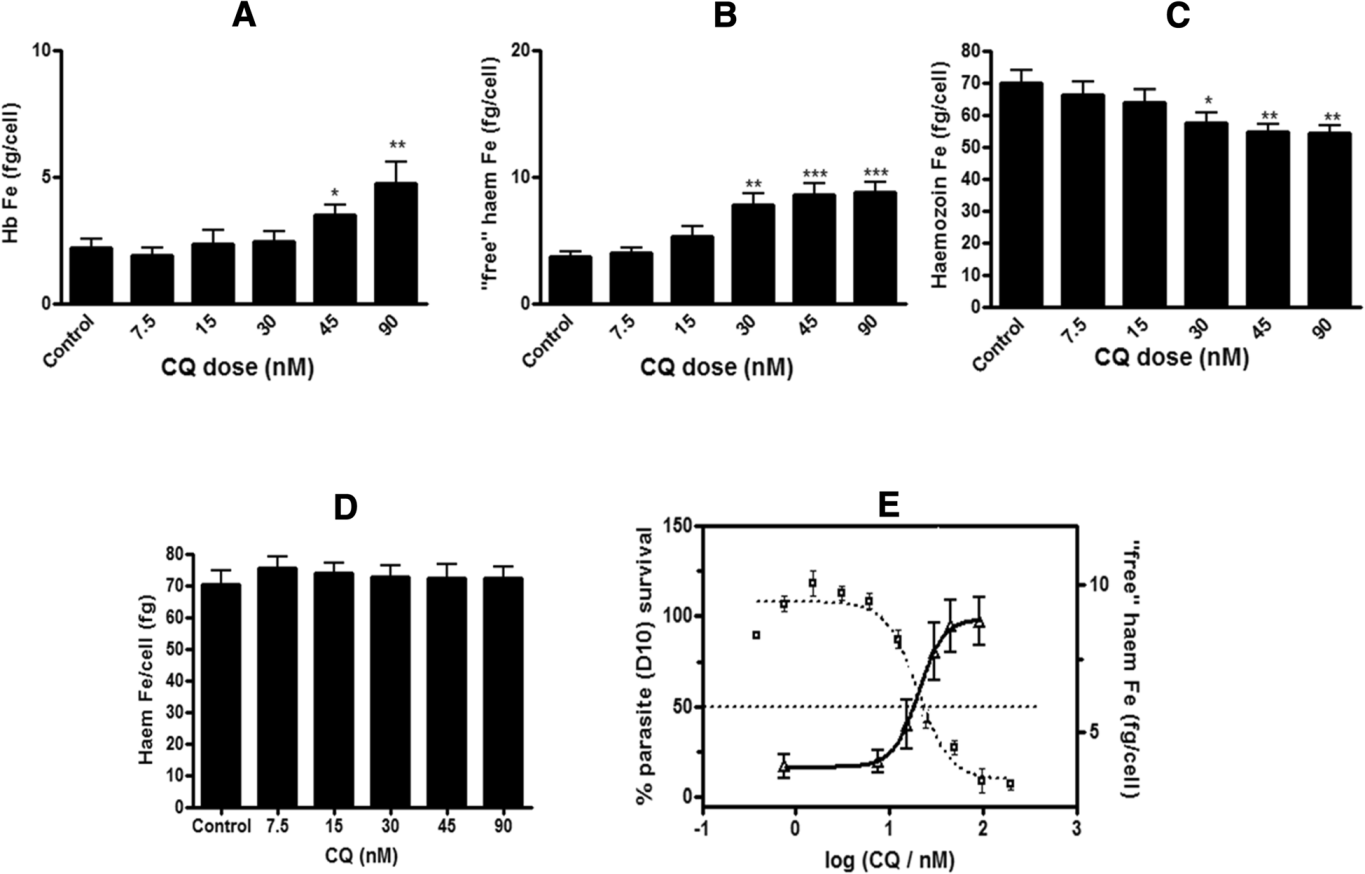
Amount of haem Fe per cell (*fg*) found in chloroquine-treated trophozoites (plate method). Hb (**a**), ‘free’ haem (**b**) and Hz (**c**) at increasing concentrations of CQ expressed as total amount of haem Fe per isolated trophozoite (*fg/cell*). Total haem Fe per cell in the control was not statistically different to untreated cells (**d**). Statistical significance, calculated using a two-tailed *t*-test (*error bars* showing 95 % CI) is expressed relative to the control using *asterisks*. The parasite survival curve was determined using the parasite lactate dehydrogenase assay [22] and is overlaid with the total amount of ‘free’ haem Fe per isolated trophozoite as a function of CQ concentration (**e**). CQ IC_50_ in D10 = 19.2 ± 4.2 nM

Cell counts determined by flow cytometry were used to determine the amount of haem Fe per trophozoite (fg/cell) at each dose of CQ. Previous studies on trophozoites isolated using the older flask method with haemocytometer cell counting showed that the total amount of haem Fe in CQ-treated cells was not statistically different to control cells [12]. These results were confirmed in the current study where it was found that the total haem Fe in CQ-treated trophozoite cells was statistically indistinguishable from control cells (Fig. 8d). The haem fractionation profiles showing the amount of Hb (Fig. 8a), ‘free’ haem (Fig. 8b) and Hz (Fig. 8c) Fe per trophozoite with increasing CQ concentration followed the same trend as the corresponding percentage profiles seen in Fig. 6d, e and f. Similarly an overlay of the parasite survival curve with the amount of ‘free’ haem Fe per trophozoite shows an increase in the amount of ‘free’ haem per cell correlated to cell death (Fig. 8e) with both graphs intersecting at a point corresponding to 50 % parasite survival.

### Amodiaquine

**Fig. 9:**
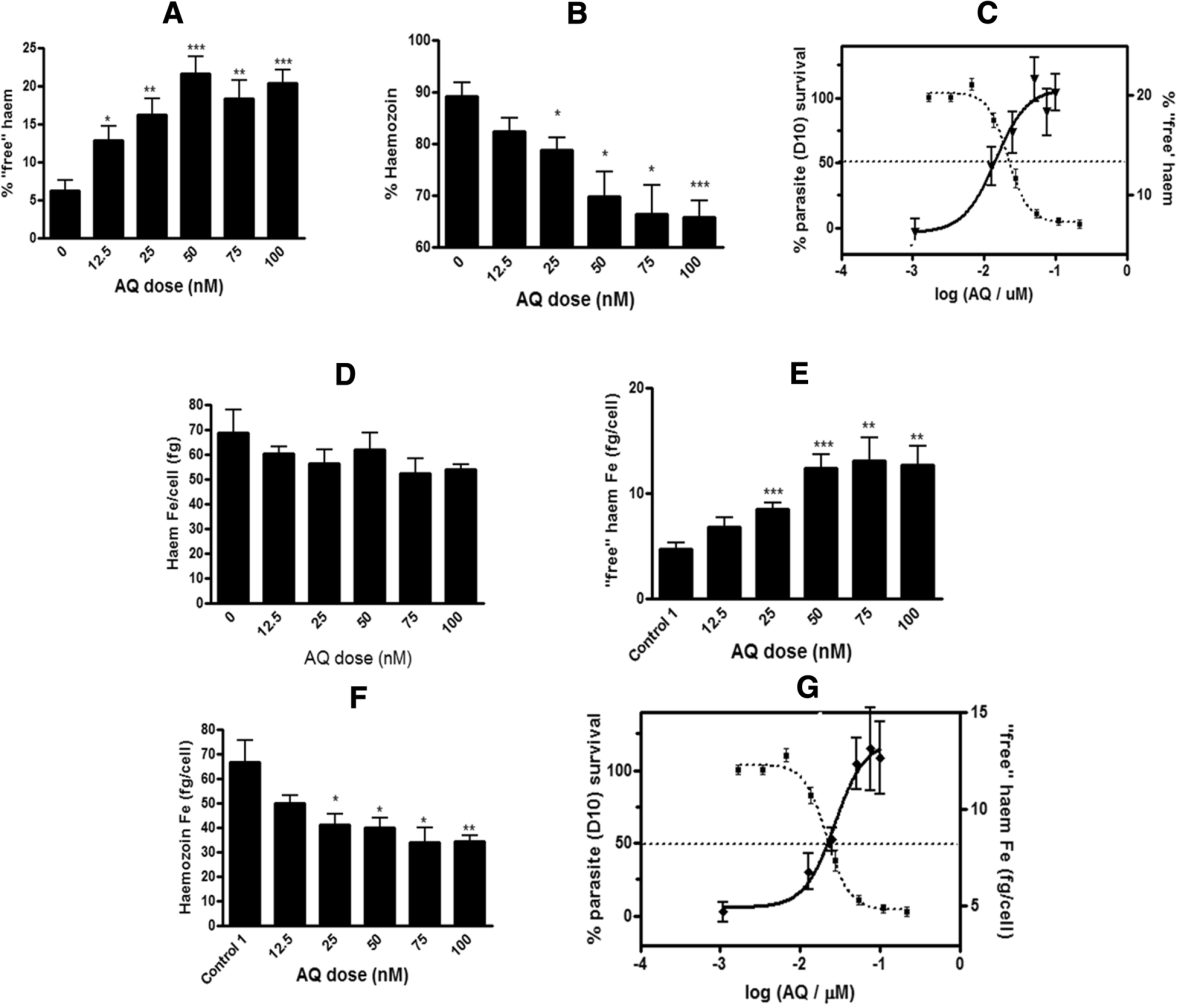
Haem fractionation profiles for amodiaquine-treated parasites. The percentages of ‘free’ haem (**a**) and Hz (**b**) are shown at increasing concentrations of AQ. The parasite survival curve for AQ, determined using the parasite lactate dehydrogenase assay [22] overlaid with the percentage of ‘free’ haem shows a rise in ‘free’ haem with increasing AQ concentration corresponding to decreased parasite survival (**c**). The total haem Fe per trophozoite of all AQ-treated cells is not statistically different to untreated cells (**d**). The amount of ‘free’ haem Fe (**e**) and Hz Fe (**f**) are shown at increasing concentrations of AQ. The parasite survival curve for AQ, overlaid with the amount of ‘free’ haem shows a rise in ‘free’ haem with increasing AQ concentration correlated to decreased parasite survival (G). AQ IC_50_ in D10 = 21.6 ± 3.0 nM

The new method was applied to another 4-aminoquinoline, AQ, which like CQ has also been shown to inhibit BH formation with the NP-40 detergent mediated BH inhibition assay [7]. Preliminary results for ‘free’ haem performed on parasites treated with AQ at 2.5× the parasite growth inhibition IC_50_ showed an increase in ‘free’ haem significantly different to the control [12]. A full dose response carried out using the plate method confirmed that AQ, like CQ was a Hz inhibitor causing a dose related increase in ‘free’ haem accompanying parasite death (Fig. 9a, b and c). No statistical difference was found in total haem Fe/trophozoite (fg/cell) between the control and AQ-treated samples (Fig. 9d). This result made it possible to use the percentage values of ‘free’ haem (Fig. 9a) and Hz (Fig. 9b) as a direct indication of the effect of AQ on each of the haem species, as was the case for CQ (Figs. 6 and 7). The profiles for the amount of ‘free’ haem Fe/trophozoite and Hz Fe/trophozoite with increasing AQ concentration (Fig. 9e and f) mirror the profiles obtained with the percentage values of the corresponding haem species (Fig. 9a and b).

### Pyrimethamine

**Fig. 10:**
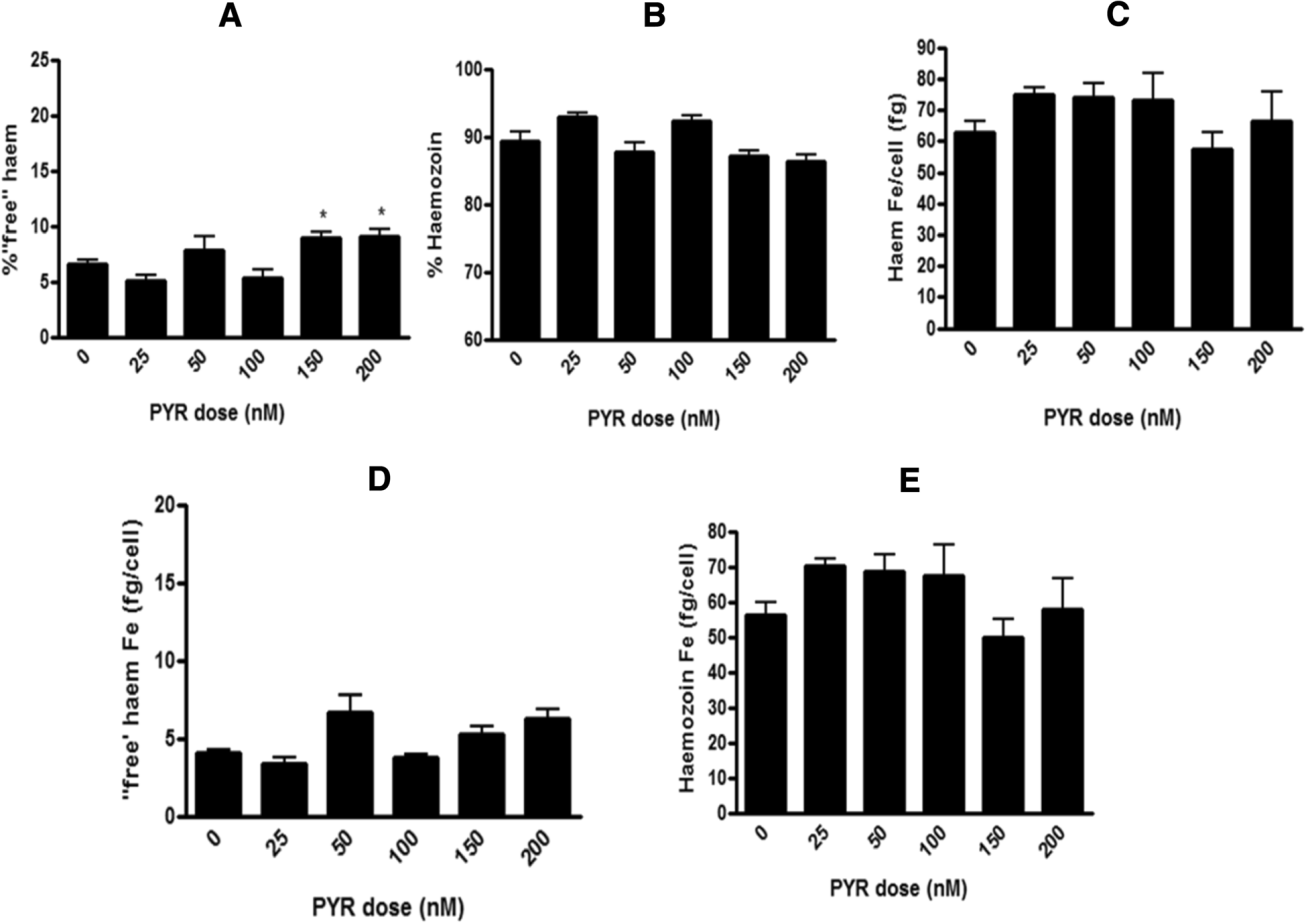
Haem fractionation profiles for pyrimethamine. Percentages of ‘free’ haem (**a**) and Hz (**b**) are shown at increasing concentrations of PYR. No statistical difference exists between the total haem Fe per cell of all PYR treated cells and untreated cells (**c**). The amount of ‘free’ haem Fe (**d**) and Hz Fe (**e**) are shown at increasing concentrations of PYR. PYR IC_50_ as determined in D10 = 44 ± 6.8 nM

The plate method was applied to the non-BH inhibiting antifolate PYR [7]. Previously used as a negative control, PYR showed no increase in ‘free’ haem or decrease in Hz when tested at 2.5× its parasite growth inhibition IC_50_ [12]. At higher concentrations of PYR corresponding to 3.5× and 4.5× IC_50_, the per cent ‘free’ haem (Fig. 10a) does show a statistically significant increase compared to the control. This increase in ‘free’ haem is however not dose related and is not accompanied by a decrease in per cent Hz (Fig. 10b) which remains constant at all PYR concentrations. Furthermore, the increase in the amount of ‘free’ haem is significant only at the highest dose. These data are consistent with the fact that that PYR is not a Hz inhibitor.

### Atovaquone

The method was also applied to Atov which shows no BH inhibition, but has a mechanism of action known to selectively disrupt mitochondrial electron transport in the parasite by inhibiting the cytochrome *bc*_*1*_ complex without affecting host cell mitochondria [25, 26].

**Fig. 11:**
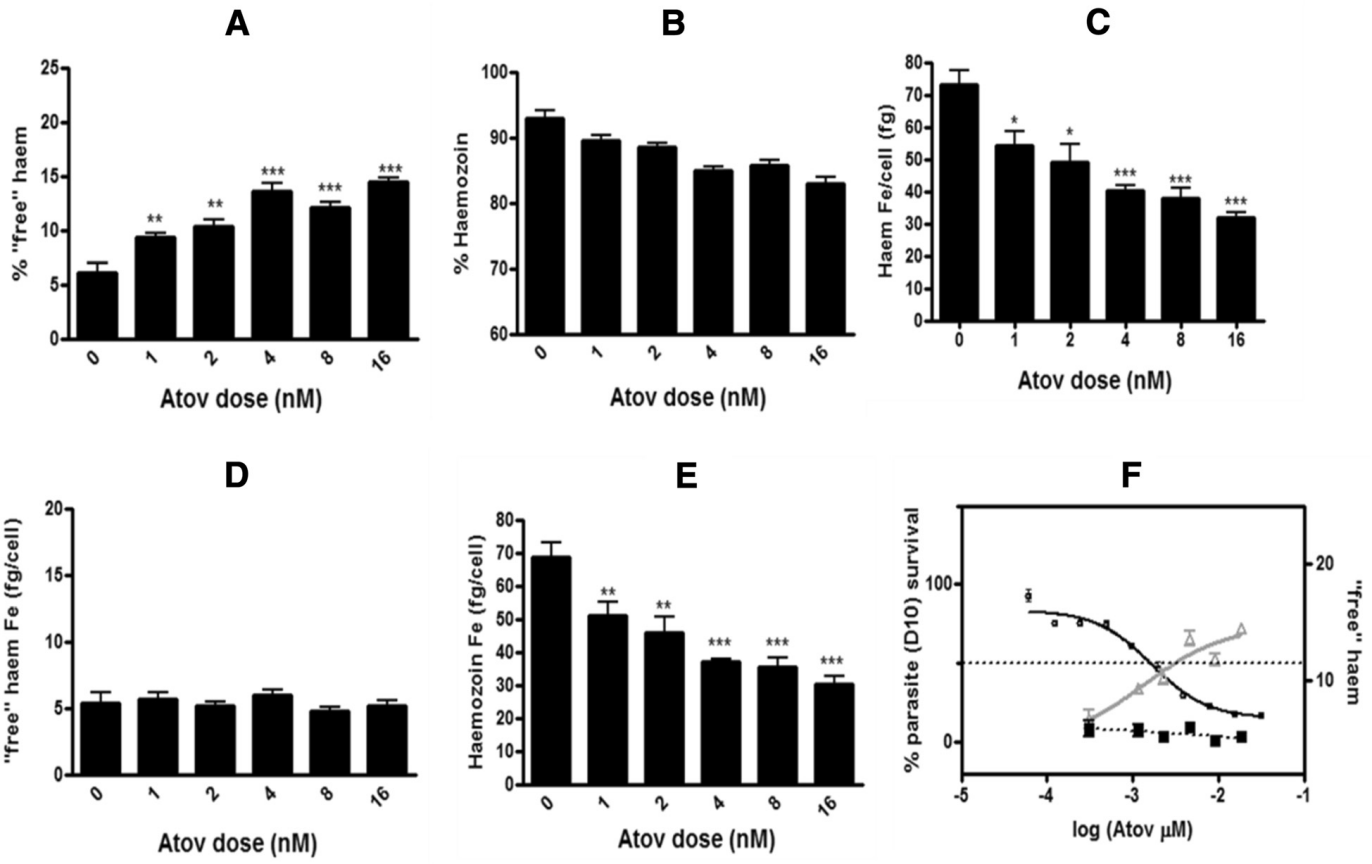
Haem fractionation profiles for atovaquone. Increase in percentage ‘free’ haem (**a**) and decrease in percentage Hz (**b**) seen with increasing Atov concentration. The total haem Fe (*fg*) per trophozoite of Atov-treated cells is statistically different to untreated cells (**c**). Graphs **d** and **e** show the amount of ‘free’ haem Fe (*fg/cell*) and Hz Fe per trophozoite respectively with increasing Atov concentration. The parasite survival curve for Atov overlaid with the percentage of ‘free’ haem (*grey* graph with *open triangles*, right axis) shows an apparent rise in ‘free’ haem with increasing Atov concentration correlated to parasite survival (**f**). However, by contrast to Hz inhibitors CQ and AQ, no relationship in fact exists between parasite survival and the actual amount of ‘free’ haem Fe (fg/cell) per trophozoite (*black dashed* graph with *closed squares*, right axis) which remains constant at 5.4 ± 0.4 fg haem Fe per cell. Atov IC_50_ as determined in D10 = 2.0 ± 0.8 nM

Surprisingly, Atov clearly showed a significant dose dependent increase in percentage ‘free’ haem and decrease in percentage Hz (Fig. 11a, b). Although the increase was less than that seen for both CQ and AQ at equivalent IC_50_ values, it was nonetheless significant and dose related. Further investigation however, revealed decreasing total haem Fe per trophozoite in Atov-treated cells, showing that treated cells accumulated less haem than control cells (Fig. 11c). This is in contrast to results obtained for total haem Fe per trophozoite in both CQ (Fig. 7d), AQ (Fig. 9d) and PYR (Fig. 10c) treated cells, where cells treated with drug showed no significant difference to untreated cells. Despite the percentage increase in ‘free’ haem with increasing Atov concentration, when the amount of ‘free’ haem Fe per trophozoite (fg/cell) was calculated, it was found to remain constant with increasing dose, with no significant difference to the control (5.4 ± 1.6 fg Fe/cell), at the 95 % CI. The decrease in Hz is likely a result of decreased Hb uptake and digestion secondary to treatment with this drug. This result does, however, demonstrate the importance of determining the total haem Fe per cell, using cell counts determined with either a haemocytometer or FACS. In the case of Atov, where the total haem Fe in treated cells was significantly less than the control (Fig. 11c), the percentage values for each haem species are misleading (Fig. 11a and b) and the amounts of each haem species per cell (Fig. 11d and e) must be used. This approach demonstrated that there was no change in ‘free’ haem and no relationship with inhibition of parasite growth (Fig. 11f).

## Conclusion

The successful adaptation of the haem fraction assay from a very low throughput method with high demand on valuable parasite starting material to a more efficient process was achieved and validated using CQ as a model. The improved throughput plate method is capable of reproducing the results obtained with the flask method using 24 times less starting material and increasing output by at least six-fold. A fluorescent flow cytometry-based technique for the determination of cell counts of isolated trophozoites was also developed. This new method of determining cell counts provided a faster alternative to traditional manual methods of cell counting. The plate method was subsequently applied to several other established anti-malarials, providing rapid and valuable insight into mechanistic action of anti-malarials and has highlighted the important role which haem plays in the action of several drugs. Results with Atov emphasize the crucial importance of measuring haem levels per cell rather than just percentages of haem species in order to interpret the effects of drugs on parasite haem concentrations. This approach is essential in those cases where the total haem iron per cell changes with drug dose. Applied to novel compounds, this method will provide an important in vitro complement to the existing detergent-mediated BH inhibition assay in improving understanding of mechanism of action and in exploring structure activity relationships and prospects for rational drug design.

## Abbreviations

CQ: chloroquine
Hz: haemozoin
Hb: haemoglobin
TEM: transmission electron microscopy
EELS: electron energy loss spectroscopy
BH: β-haematin
CQS: chloroquine sensitive strain
DMSO: dimethyl sulfoxide
PBS: phosphate buffered saline
FACS: flow assisted cell sorting
FSC: forward scatter
SSC: side scatter
AQ: Amodiaquine
Atov: atovaquone
PYR: pyrimethamine
